# A Vision Based Detection Method for Narrow Butt Joints and a Robotic Seam Tracking System

**DOI:** 10.3390/s19051144

**Published:** 2019-03-06

**Authors:** Boce Xue, Baohua Chang, Guodong Peng, Yanjun Gao, Zhijie Tian, Dong Du, Guoqing Wang

**Affiliations:** 1 Department of Mechanical Engineering, Tsinghua University, Beijing 100084, China; xbc17@mails.tsinghua.edu.cn (B.X.); bhchang@tsinghua.edu.cn (B.C.); pgd16@mails.tsinghua.edu.cn (G.P.); 2 Key Laboratory for Advanced Materials Processing Technology, Ministry of Education, Beijing 100084, China; 3 Capital Aerospace Machinery Ltd., Beijing 100076, China; gaoyanjun274507@163.com (Y.G.); tzhj_2004@126.com (Z.T.)

**Keywords:** robotic welding, seam tracking, visual detection, narrow butt joint, GTAW

## Abstract

Automatic joint detection is of vital importance for the teaching of robots before welding and the seam tracking during welding. For narrow butt joints, the traditional structured light method may be ineffective, and many existing detection methods designed for narrow butt joints can only detect their 2D position. However, for butt joints with narrow gaps and 3D trajectories, their 3D position and orientation of the workpiece surface are required. In this paper, a vision based detection method for narrow butt joints is proposed. A crosshair laser is projected onto the workpiece surface and an auxiliary light source is used to illuminate the workpiece surface continuously. Then, images with an appropriate grayscale distribution are grabbed with the auto exposure function of the camera. The 3D position of the joint and the normal vector of the workpiece surface are calculated by the combination of the 2D and 3D information in the images. In addition, the detection method is applied in a robotic seam tracking system for GTAW (gas tungsten arc welding). Different filtering methods are used to smooth the detection results, and compared with the moving average method, the Kalman filter can reduce the dithering of the robot and improve the tracking accuracy significantly.

## 1. Introduction

In automatic welding, it is necessary to align the welding torch with the center of the joint to ensure the welding quality. Nowadays, the motion path of a welding robot is usually set by offline programming or manual teaching. However, during the welding process, the actual joint trajectory may deviate from the path set before welding due to factors, such as machining error, assembly error, and thermal deformation. In view of the abovementioned reason, it is necessary to perform automatic joint detection.

In the welding field, visual detection is widely used for the monitoring of weld defects [1], recognition of the weld joint [2,3,4], etc. For automatic joint detection, the structured light method based on optical triangulation is commonly used to detect the 3D position of joints with large grooves. Zou et al. projected a structured laser on the workpiece surface and extracted laser stripes from images strongly disturbed by an arc to calculate the 3D position of the joint in the world frame and control the motion of the welding torch in real time [5]. Li et al. proposed a robust automatic welding seam identification and tracking method by utilizing structured light vision, which can identify deformed laser stripes in the complex welding environment and find the position of the welding joint in the pixel coordinate [6]. Some companies have released commercial joint detection sensors based on the structured light method [7,8].

However, for the butt joint with a narrow gap (with a width less than 0.2 mm), the deformation of structured light stripes almost disappears, so it is difficult to detect the position of the narrow butt joint with the structured light method [9]. To solve this problem, researchers have proposed a variety of methods. Xu et al. developed a passive visual sensing method to capture the image of a molten pool and extracted the edge of the molten pool with an improved Canny operator to calculate the deviation of the joint relative to the torch [10]. Gao et al. tried to capture the image of a molten pool with an infrared camera and calculated the deviation of the joint relative to the torch by the shape of the weld pool. Then, an adaptive Kalman filter and Elman neural network were used to improve the accuracy [11]. Nilsen et al. estimated the offset of the joint relative to the torch in laser welding by the image of the keyhole and the spectrum of the plasma sprayed from the keyhole, respectively, and combined these two methods to construct a composite sensing system [12]. Shah et al. used an auxiliary light source to illuminate the workpiece. Considering the uneven brightness on the surface of the workpiece, the local thresholding method was used to extract the position of the joint in pixel coordinates [13]. Nele et al. constructed an image acquisition system, which was combined with the pattern learning algorithm to detect the position of the butt joint relative to the torch and corrected the torch position in real time [14]. Kramer et al. distinguished the boundaries between the two surfaces of the workpiece to be welded by their texture information, thereby finding the nearly invisible narrow line imaged by the joint gap [15]. Gao et al. introduced a novel method, in which the deviation of the weld joint relative to the torch was detected according to the magneto-optical effect [16].

The above methods for the detection of a narrow butt joint can only detect its 2D position. However, in the welding of a butt joint with a width less than 0.2 mm and with a 3D trajectory, the 3D position of the joint is required. Furthermore, the welding torch should also maintain a proper orientation relative to the workpiece surface to ensure the welding quality, so the normal vector of the workpiece surface also needs be obtained. Fang et al. presented a visual seam tracking system in which the deviation of the joint relative to the torch in the horizontal direction was detected according to the position of the joint in the image under natural light illumination, and the deviation in the vertical direction was detected using the structured light method, but the method was incapable of detecting the orientation of the workpiece surface [17]. Shao et al. projected three laser stripes onto the workpiece surface, blended the 2D information of the joint in the image with the 3D information of the structured light, calculated the 3D position of the joint and the normal vector of the workpiece surface, and adjusted the position and orientation of the torch in real time [18]. However, this method still relied on the deformation of laser stripes. Because of the machining error and assembly error, the width of the joint can be uneven and the gap of some points on the joint will disappear. Under this circumstance, this method will miss some joint points. 

Zeng et al. designed a narrow butt detection sensor, which projected uniform light and crosshair structured light onto the surface of a workpiece and captured images alternately. Then, 2D and 3D information were combined to calculate the 3D position of the joint and the normal vector of the workpiece surface in a world frame, and the position of the torch was corrected in real time [9,19]. Based on this method, Peng et al. tried to fit the workpiece surface with the moving least squares (MLS) method in the calculation process to improve the fitting accuracy [20]. However, Zeng’s method [9,19] has certain limitations. To conveniently extract the 2D information of the joint from the image, the method requires strict lighting conditions for the auxiliary light source. When the auxiliary light source is on, the grayscale of the workpiece surface in the image needs to be almost saturated, but this requirement can be achieved only with a specular reflection workpiece surface and the workpiece surface needs to be close to the auxiliary light source. The working distance of those commercial joint detection sensors based on the structured light method can reach more than 100 mm [7,8], while the working distance in [9] does not exceed 40 mm generally. In the case of a diffuse reflection workpiece surface or remote detection, the illumination intensity of the LED light source is insufficient as the auxiliary light source. To achieve the desired high grayscale of the workpiece surface in the image, the exposure time of the camera needs to be extended, which will deteriorate the detection speed. If a laser light source is used as the auxiliary light source, speckle in the image will affect the image quality and make it difficult to extract the 2D information of the joint from the image. In this paper, a vision based detection method for a narrow butt joint is proposed, which reduces the requirements for the lighting conditions of the auxiliary light source, and the proposed method is used in building a robotic seam tracking system for GTAW (gas tungsten arc welding).

The rest of the paper is organized as follows. In Section 2, the processes and details of the proposed detection method are presented. To apply this detection method in the robotic seam tracking system, Section 3 introduces the necessary coordinate transformation. In Section 4, the configurations of the joint detection sensor and the robotic seam tracking system for GTAW are detailed. In Section 5, the detection results of the proposed method are presented, and different filtering methods are used to smooth the detection results to reduce the dithering of the robot and improve the seam tracking accuracy. Finally, Section 6 gives the conclusions of this paper.

## 2. Detection Method for the Narrow Butt Joint

In this section, the principle of the detection method is introduced first, then details of the method are discussed, including the grabbing of images with an appropriate grayscale distribution, image processing, and the calculation of the position and orientation of the joint. Finally, applications of the proposed method are discussed.

### 2.1. Principle of the Method

**Figure 1:**
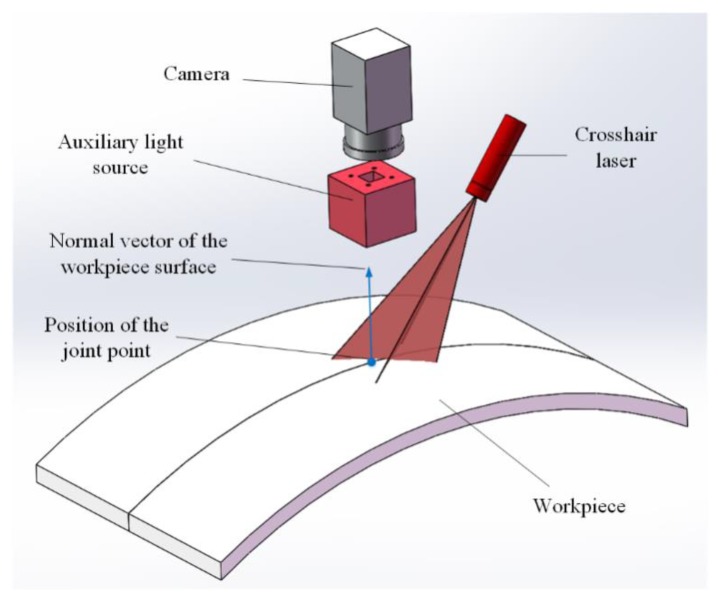
Basic experimental setup of the proposed method.

**Figure 2:**
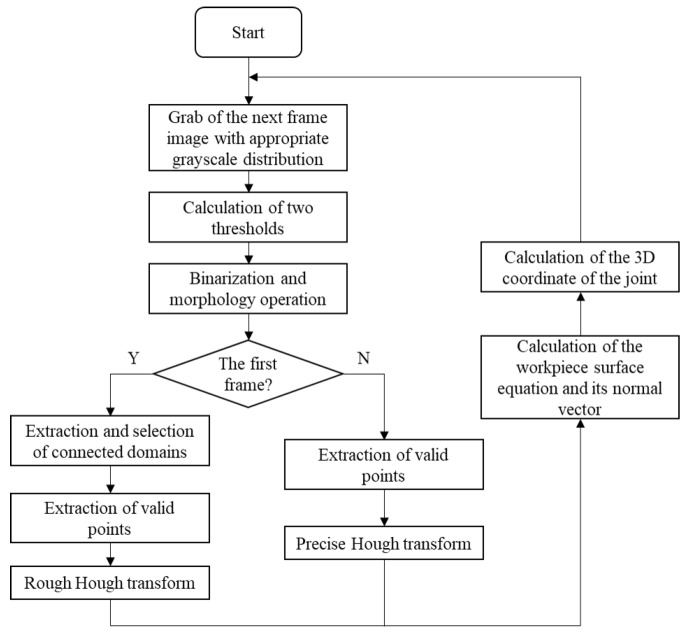
Flowchart of the proposed joint detection method.

The basic experimental setup of the proposed detection method for a narrow butt joint is shown in Figure 1. A crosshair laser is projected onto the workpiece surface and the LED (light-emitting diode) auxiliary light source is used to illuminate the workpiece surface continuously. Images with an appropriate grayscale distribution are grabbed by using the auto exposure function of the camera to adjust the exposure time. Then, the joint region and laser stripe region can be extracted by different gray thresholds. The laser stripe region provides the 3D information of the workpiece surface (normal vector included) and the joint region provides the 2D information of the joint. By combining the 2D and 3D information together, the 3D position of the joint can be obtained. To improve the processing speed, different processing flows are used for the first frame and the subsequent frames. The flowchart of the proposed joint detection method is shown in Figure 2, and its details will be described in the following subsections.

### 2.2. Grabbing of Images with an Appropriate Grayscale Distribution

**Figure 3:**
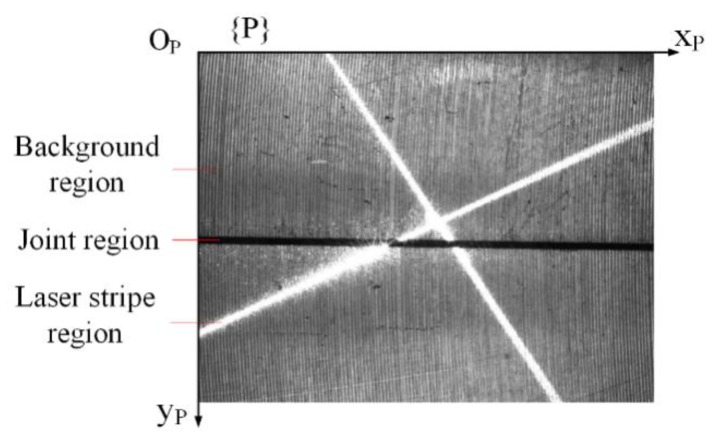
The grabbed ROI (region of interest).

The ROI (region of interest) of the grabbed image is shown in Figure 3, and the field-of-view corresponding to it is 16.25 × 10 mm. The grayscale of the background region is affected by the illumination intensity of the auxiliary light source. The laser stripe region is created by the projection of the crosshair laser. Ideally, the grayscale for the joint region should be very low (close to 0) and that for the laser stripe region should be very high (close to 255) in the image. So, in the auto exposing of the camera, the expected average grayscale of the ROI is set to 128 to ensure that the joint region and laser stripe region can be differentiated clearly from the background region according to different gray thresholds. However, if the illumination intensity of the auxiliary light source is too strong, the exposure time will be reduced greatly, so the grayscale for the laser stripe region will be obviously smaller than 255 and be close to that of the background region when the expected average grayscale of the ROI is set to 128, which will make it difficult to differentiate the laser stripe region from the background region. So, to differentiate the laser stripe region from the background region, the illumination intensity of the auxiliary light source is controlled to be obviously weaker than that of the crosshair laser by adjusting its supply voltage to make sure that the grayscale for the laser stripe region is close to 255. In fact, this can easily be achieved, especially under the circumstance of remote detection, because the orientation of the LED light is much worse than that of the laser. The average grayscale of the ROI shown in Figure 3 is 128.4. The pixel coordinate system {P} is established on the ROI.

### 2.3. Image Processing

#### 2.3.1. Determination of Thresholds for Binarization

**Figure 4:**
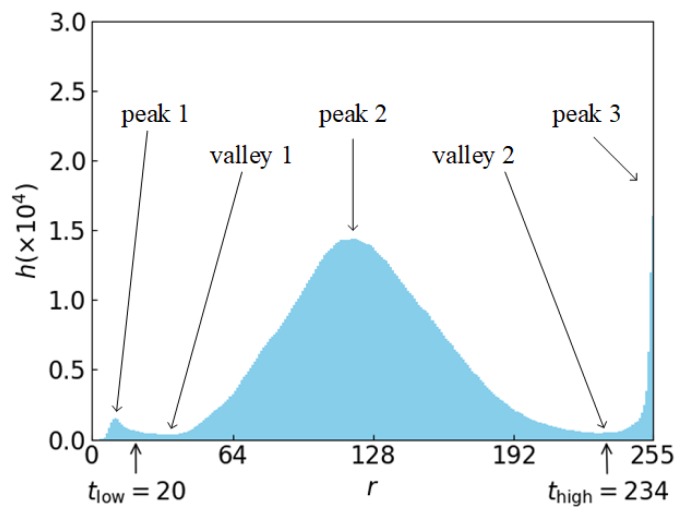
Smoothed histogram of the grabbed ROI.

The thresholds for the extraction of the laser stripe region and the joint region are determined according to the histogram, h(r), of the ROI, where r represents the grayscale and h represents the number of pixels with a grayscale of r. To eliminate false valleys caused by accidental factors, a Gaussian filter with a length of 5 is used to smooth the original histogram first, and the smoothed histogram is shown in Figure 4. The background region with a medium grayscale results in peak 2 in the histogram. For the laser stripe region, its grayscale is very high and its area is not very small, which causes peak 3 in the high grayscale region of the histogram. So, the grayscale corresponding to valley 2, which is between peak 2 and peak 3, can be regarded as the threshold, $t_{\mathrm{high}}$, and it can be used for extracting the laser stripe region from the background region. The grayscale corresponding to valley 2 is found to be 234 in the smoothed histogram, so $t_{\mathrm{high}}=234$.

For the joint region, the binarization threshold cannot be determined in the same way as the laser stripe region. This is because when the joint is extremely narrow, the area of the joint region in the ROI can be very small. In this case, peak 1 in the histogram does not exist at all, and neither does valley 1. To ensure the robustness of the threshold determination method, Equation (1) is used to determine the threshold, $t_{\mathrm{low}}$, for extracting the joint region from the background region:

(1) $$\begin{array}{l} t_{\mathrm{low}}=\max r\\ s,t.\;\overset{r}{\underset{i=0}{\sum }}h\left(i\right)\leq Sa \end{array}$$

where S is the area of the ROI and a is a ratio, which is a=0.01. We get $t_{\mathrm{low}}=20$. The value of a should be near the percentage of the joint region’s area in the ROI. If the value is set too high, the background region is not eliminated effectively. On the contrary, if the value is set too low, the main part of the joint region is not kept completely. 

#### 2.3.2. Binarization and Morphology Operation

**Figure 5:**
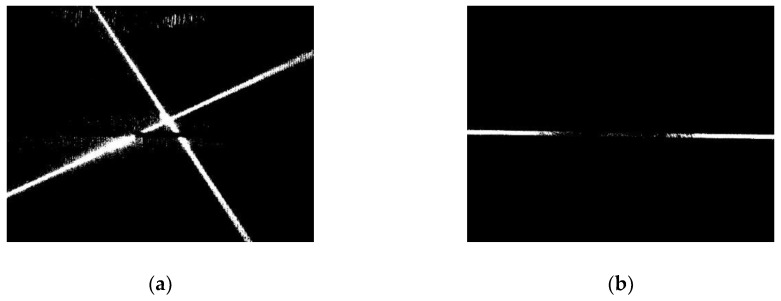
ROI after binarization. (**a**) Laser stripe region. (**b**) Joint region.

**Figure 6:**
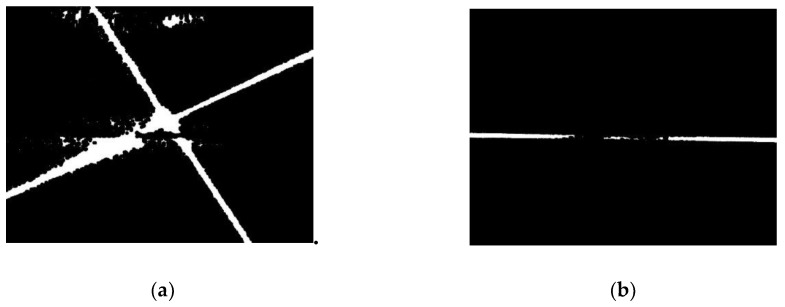
ROI after close operation. (**a**) Laser stripe region. (**b**) Joint region.

The binarization of the images is processed with the thresholds, $t_{\mathrm{high}}$ and $t_{\mathrm{low}}$, respectively, and the binary ROI are shown in Figure 5. In the two images of Figure 5, there are some disconnected small regions, so the morphology of the close operation is used to connect them in the two images, and the images after close operation are shown in Figure 6.

#### 2.3.3. Extraction and Selection of Connected Domains

When the image is the first frame, the connected domains in the two images of Figure 6 are extracted. Because the laser stripe region and the joint region, ideally, do not have holes, only the outermost contours are kept if there are nested contours. For the laser stripe region and joint region, the connected domains have a relatively large area and a slender shape, while those falsely kept regions always have a relatively small area or a less slender shape, so we can retain the laser stripe region and joint region according to their area and circularity ratio [21] (pp. 844–845):

(2) $$\{\begin{array}{c} A>A_{\min}\\ R_{c}<R_{\mathrm{cmax}} \end{array}$$

where A is the area of the connected domains and $A_{\min}$ is the area threshold. The values of $A_{\min}$ are selected by several attempts to keep the main part of the laser stripe region or the joint region. For the laser stripe region, $A_{\min}$ is set to 2000 pixel^2^ and for the joint region, $A_{\min}$ is set to 1000 pixel^2^. This is because the area of the laser stripe region is obviously larger than that of the joint region. $R_{c}$ represents the circularity ratio of the connected domains and $R_{\mathrm{cmax}}$ is the circularity ratio threshold. $R_{c}$ is defined as:

(3) $$R_{c}=\frac{4\pi A}{P^{2}}$$

where P is the perimeter of the connected ratio. The circularity ratio can represent the slenderness degree of a region. It is 1 for a circular region and 0 for a line, so the circularity ratio threshold, $R_{\mathrm{cmax}}$, is set to 0.5 for both the laser stripe region and the joint region and we find this value effective.

**Figure 7:**
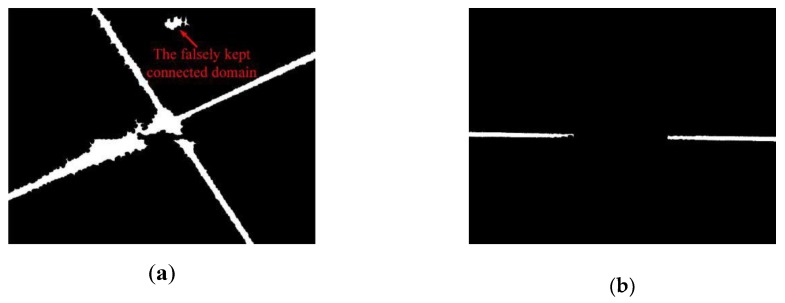
Kept connected domains. (**a**) Laser stripe region. (**b**) Joint region.

The connected domains kept are shown in Figure 7. It can be found that there is a false connected domain kept in Figure 7a, which results from the area threshold being not large enough. In fact, if the area threshold is set to 3000 pixel^2^, this false connected domain will be eliminated. However, even if it is kept, the laser stripe can still be extracted successfully in the following steps.

Because the extraction and selection of the connected domains are time-consuming tasks, to improve the speed of image processing, they are only performed for the first frame. For the rest of the frames, those falsely kept regions do not influence the detection result because of the robustness of the image processing method, which is introduced in the content below.

#### 2.3.4. Extraction of Valid Points

**Figure 8:**
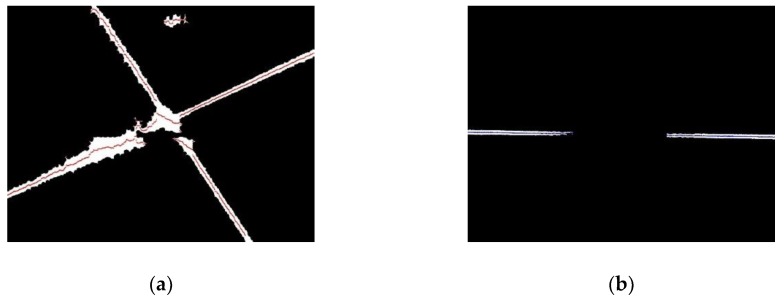
Extracted valid points. (**a**) Laser stripe region. (**b**) Joint region.

For the images in Figure 7, each column is scanned from top to bottom to find every line segment whose length is greater than 10, and then the midpoint of each found line segment is marked as a valid point, as shown in Figure 8. The valid points of the laser stripe region are marked in red and the valid points of the joint region are marked in blue. The use of a length threshold of the line segments is to eliminate those line segments located at the boundary of the connected domains.

#### 2.3.5. Line Extraction

Because the field-of-view corresponding to the ROI is small (with a size of 16.25 × 10 mm), the joint in the ROI can be approximated as a straight line when its trajectory does not change sharply. When the curvature of the joint trajectory increases, the size of the field-of-view corresponding to the ROI should be reduced to make sure that the joint region can be regarded as a straight line. Similarly, the workpiece surface in the ROI can be approximated as a plane when its curvature is small, so the laser stripes can be approximated as two straight lines.

The Hough transform [22] is a common method for the detection of straight lines. A line can be represented as ρ=xcosθ+ysinθ, where ρ is the perpendicular distance from the origin to the line and θ is the angle formed by this perpendicular line and the horizontal axis, so any line can be represented with (ρ,θ). A 2D array or accumulator is created with a resolution of Δρ×Δθ. For every point in the image, θ is changed within its domain of definition with a step size of Δθ and a different ρ is obtained. For every (ρ,θ) pair, the value of the bin corresponding to it in the accumulator increases. Finally, the parameters of the bin corresponding to the maximum value in the accumulator are regarded as the extracted line.

However, the computational cost of the Hough transform increases with the improvement of its detection accuracy. To ensure the detection accuracy of the Hough transform and meanwhile ensure the speed of detection to meet the requirements of real-time performance, different parameter settings are adopted for the first frame and the subsequent frames. If the speed of detection is not fast enough, the detected point would not be dense enough so the detection accuracy of the joint trajectory will deteriorate.

**Figure 9:**
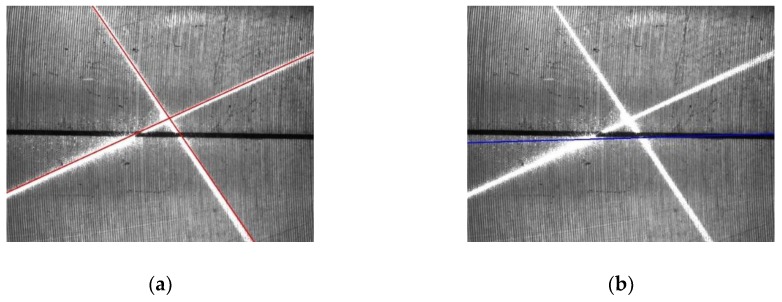
Extracted lines with the rough Hough transform. (**a**) Laser stripe lines. (**b**) Joint line.

For the first frame, a rough Hough transform is used. The Hough transform is applied to extract two laser stripe lines and the joint line, respectively, and only valid points in Figure 8 are considered. To increase the detection speed, the resolution, Δρ and Δθ, are set with relatively large values, which means that the accuracy of the line extraction is relatively low. $\Delta \rho =\Delta \rho_{f}=10\;\mathrm{pixel}$ and $\Delta \theta =\Delta \theta_{f}=0.1\;\mathrm{rad}$ are set here. Because we currently do not know any information about the line’s position, the value range of ρ is set to [−diag, diag], where diag=1640 pixel is the diagonal length of the ROI and the value range of θ is set to $\left[-\frac{\pi }{2},\;-\frac{\pi }{2}\right]$. For the valid points of the laser stripe region, the accumulator’s bins with maximum values in the range of θ>0 and θ<0 are searched, respectively, and the parameters of these two found bins represent the two laser stripe lines. For the valid points of the joint region, the accumulator’s bin with the maximum value is searched for in the whole parameter space of ρ and θ, and the parameters of the found bin represent the joint line. The found laser stripe lines and the joint line are drawn in Figure 9a,b, respectively, from which it can be seen that the accuracy of the line extraction is inadequate.

**Figure 10:**
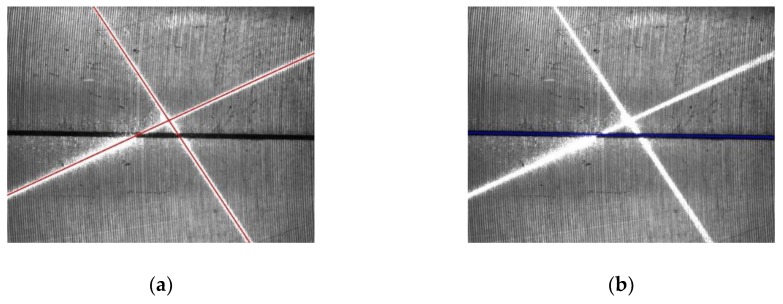
Extracted lines with a precise Hough transform. (**a**) Laser stripe lines. (**b**) Joint line.

For the subsequent frames, a precise Hough transform is used. During two successive frames, there is no significant relative motion between the camera and the workpiece, so the position of the laser stripe region and the joint region only changes a little. Therefore, from the second frame, the value ranges of the parameters of the Hough transform only need to be near the extracted lines of the last frame. For example, suppose that the parameters of the extracted joint line in one frame are $\left(\rho_{l},\theta_{l}\right)$. Then, the value of ρ is restricted to $\left[\rho_{l}-\rho_{n},\rho_{l}+\rho_{n}\right]$ and θ is restricted to $\left[\theta_{l}-\theta_{n}\;,\;\theta_{l}+\theta_{n}\right]$ when extracting the joint line in the subsequent frame. $\rho_{n}$ and $\theta_{n}$ represent half of the value range of ρ and θ in the Hough transform of the subsequent frame, which are set to be 100 pixels and 0.2 rad, respectively. In the same way, the value ranges for the extraction of two laser stripe lines are determined. Since the value ranges of the parameters become smaller, the value of Δρ and Δθ can be reduced to increase the extraction accuracy, so it is set that $\Delta \rho =\Delta \rho_{s}=1\;\mathrm{pixel}$ and $\Delta \theta =\Delta \theta_{s}=0.005\;\mathrm{rad}$. The laser stripe lines and the joint line extracted with this method are shown in Figure 10, from which we can see that the accuracy of the line extraction obviously increases.

It is worth noting that the extraction and selection of the connected domains described in Section 2.3.3 are not performed for the subsequent frames, so there may be some valid points falsely extracted. The restriction for the value ranges of the parameters of the Hough transform in the subsequent frames can eliminate the effect of these falsely extracted valid points on line extraction, which therefore increases the robustness of the line extraction method.

Below is a comparison of computational complexity between the rough Hough transform and the precise Hough transform. When the number of valid points is fixed, the computational complexity of the Hough transform is $O\left(M_{1}\right)$ and the computational complexity of searching for the accumulator’s bin with the maximum value is $O\left(M_{1}M_{2}\right)$, where $M_{1}$ and $M_{2}$ are the number of possible values for θ and ρ, respectively.

For the first frame:

(4) $$M_{1}=\frac{\pi }{\Delta \theta_{f}}\approx 63,\;M_{2}=\frac{2diag}{\Delta \rho_{f}}\approx 328$$

For the subsequent frames:

(5) $$M_{1}=\frac{2\theta_{n}}{\Delta \theta_{s}}=80,\;M_{2}=\frac{2\rho_{n}}{\Delta \rho_{s}}\approx 200$$

It can be seen that compared with the first frame, the computational complexity of the precise Hough transform for the subsequent frames does not change much though its accuracy increases significantly.

### 2.4. Calculation of the 3D Coordinates of the Joint and the Normal Vectors of the Workpiece Surface

By performing calibration in advance [23], the relationship between a point $\left(x^{P},y^{P}\right)$ in the pixel coordinate system {P} and its corresponding point $\left(x^{C},y^{C},z^{C}\right)$ in the camera coordinate system {C} is obtained as:

(6) $$\{\begin{array}{l} x^{C}=z^{C}S_{x}\left(x^{P},y^{P}\right)\\ y^{C}=z^{C}S_{y}\left(x^{P},y^{P}\right) \end{array}$$

where $S_{x}\left(x^{P},y^{P}\right)$ and $S_{y}\left(x^{P},y^{P}\right)$ represent the transformation function between {P} and {C}, which are determined by the camera itself. The equations of the two light planes of the crosshair laser source in {C} can also be obtained through calibration:

(7) $$A_{i}x^{C}+B_{i}y^{C}+C_{i}z^{C}+D_{i}=0,\;i=1,2$$

Two laser stripe lines are denoted as $l_{1}$ and $l_{2}$. N points centered on the intersection of $l_{1}$ and $l_{2}$ are selected with an equal distance on $l_{1}$ and $l_{2}$ in {P}, respectively, and these selected points are denoted as $\left(x_{ij}^{P},y_{ij}^{P}\right)$, j=1,2,…,N. N is set to 50 and the distance between two adjacent points is set to 10 pixels. For each selected point $\left(x_{ij}^{P},y_{ij}^{P}\right)$, its corresponding coordinate, $\left(x_{ij}^{C},y_{ij}^{C},z_{ij}^{C}\right)$, in {C} can be solved by combining Equations (6) and (7):

(8) $$\{\begin{array}{l} z_{ij}^{C}=\frac{-D_{i}}{A_{i}S_{x}\left(x_{ij}^{P},y_{ij}^{P}\right)+B_{i}S_{y}\left(x_{ij}^{P},y_{ij}^{P}\right)+C_{i}}\\ x_{ij}^{C}=z_{ij}^{C}S_{x}\left(x_{ij}^{P},y_{ij}^{P}\right)\\ y_{ij}^{C}=z_{ij}^{C}S_{y}\left(x_{ij}^{P},y_{ij}^{P}\right) \end{array}\;,\;i=1,2,\;j=1,2,\ldots ,50$$

Because the field-of-view corresponding to the ROI is small, the workpiece surface in the ROI can be approximated as a plane in {C} when its curvature is small, and its least-squares plane can be estimated with $\left(x_{ij}^{C},y_{ij}^{C},y_{ij}^{C}\right)$, which can be represented with Equation (9):

(9) $$A_{w}^{C}x^{C}+B_{w}^{C}y^{C}+C_{w}^{C}z^{C}+D_{w}^{C}=0$$

Naturally, the normal vector of the workpiece surface in {C} can be represented as $n_{w}^{C}={\left[A_{w}^{C},\;B_{w}^{C},\;C_{w}^{C}\right]}^{T}$, which can also be regarded as the normal vector (or orientation) of the joint point.

Among the points on the joint line, only the point located at the middle along the y direction of the ROI is selected and calculated as the joint point here, which is denoted as $\left(x_{s}^{P},y_{s}^{P}\right)$ in {P}. Then, its corresponding 3D coordinate, ($x_{s}^{C},y_{s}^{C},z_{s}^{C})$, in {C} can be solved by combining Equations (6) and (9):

(10) $$\{\begin{array}{l} z_{s}^{C}=\frac{-D_{w}^{C}}{A_{w}^{C}S_{x}\left(x_{s}^{P},y_{s}^{P}\right)+B_{w}^{C}S_{y}\left(x_{s}^{P},y_{s}^{P}\right)+C_{w}^{C}}\\ x_{s}^{C}=z_{s}^{C}S_{x}\left(x_{s}^{P},y_{s}^{P}\right)\\ y_{s}^{C}=z_{s}^{C}S_{y}\left(x_{s}^{P},y_{s}^{P}\right) \end{array}\;.$$

### 2.5. Applications of the Proposed Detection Method

The proposed detection method can be used both before welding and during welding. On the one hand, it can be used before welding to correct the path of the robot when the trajectory of the joint changes after teaching. Under this circumstance, it can be applied to welding methods, like laser welding, GMAW (gas metal arc welding), and GTAW. On the other hand, it can be used during welding to guide the motion of the torch in real time. Since this method requires images of the joint with little disturbance, it can be applied to welding methods that include almost no spatter, like GTAW. 

## 3. Coordinate Transformation

In Section 2, the position and orientation of the joint point in the camera coordinate system {C} was calculated with the proposed joint detection method. When applying this method in the robotic seam tracking system, the position and orientation in the camera coordinate system {C} need to be transformed into the base coordinate system {B} of the robot to guide the motion of the robot.

**Figure 11:**
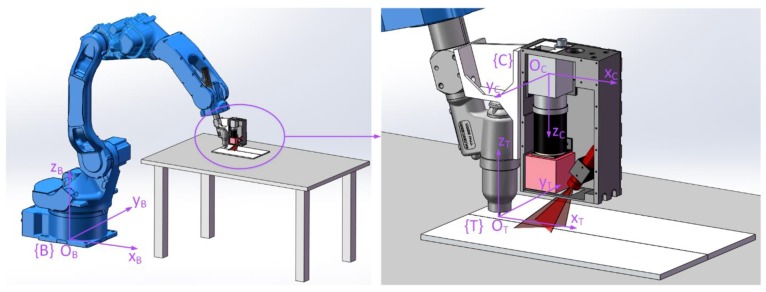
Coordinate systems involved in the coordinate transformation.

Coordinate systems involved in coordinate transformation include the camera coordinate system {C} fixed to the camera, the base coordinate system {B} attached to the robot base, and the tool coordinate system {T} fixed to the welding torch, as shown in Figure 11. A coordinate transformation can be described with a homogenous transformation matrix [24]. To describe the transformation relationship of {C} with respect to {B}, a homogenous transformation matrix, TCB, is needed. 

To obtain a homogenous transformation matrix, TCB, a homogenous transformation matrix, TCT, is required, which describes the transformation relationship of {C} with respect to {T}, and TTB, which describes the transformation relationship of {T} with respect to {B}. Then, TCB can be derived from:

(11) TCB=TTBTCT

Since in the robotic seam tracking system the camera is fixed on the welding torch, TCT can be predetermined by calibration. The origin of {T} is denoted as TCP (tool center point). TTB is related to the position and orientation of the robot, which can be represented as $\left(x_{T}^{B},y_{T}^{B},z_{T}^{B},\alpha_{T}^{B},\beta_{T}^{B},\gamma_{T}^{B}\right)$, where $\left(x_{T}^{B},y_{T}^{B},z_{T}^{B}\right)$ represents the position of the TCP in {B} and $\left(\alpha_{T}^{B},\beta_{T}^{B},\gamma_{T}^{B}\right)$ represents the orientation of the welding torch in Euler angles. $\alpha_{T}^{B}$, $\beta_{T}^{B}$, and $\gamma_{T}^{B}$ are the roll, pitch, and yaw angles of {T} relative to {B}. With these six parameters known, TTB can be derived, which is not detailed here. Additionally, TCB can be derived from Equation (11). Then, the coordinates of the joint point, $\left(x_{s}^{B},y_{s}^{B},z_{s}^{B}\right)$, in {B} can be derived from:

(12) [xsBysBzsB1]=TCB[xsCysCzsC1] .

With TCB derived, the normal vector of the joint point in {B} $n_{w}^{B}$ can be derived since $n_{w}^{C}$ is known. Suppose that $n_{w}^{B}$ can be represented as:

(13) $$n_{w}^{B}={\left[A_{w}^{B},\;B_{w}^{B},\;C_{w}^{B}\right]}^{T}$$

**Figure 12:**
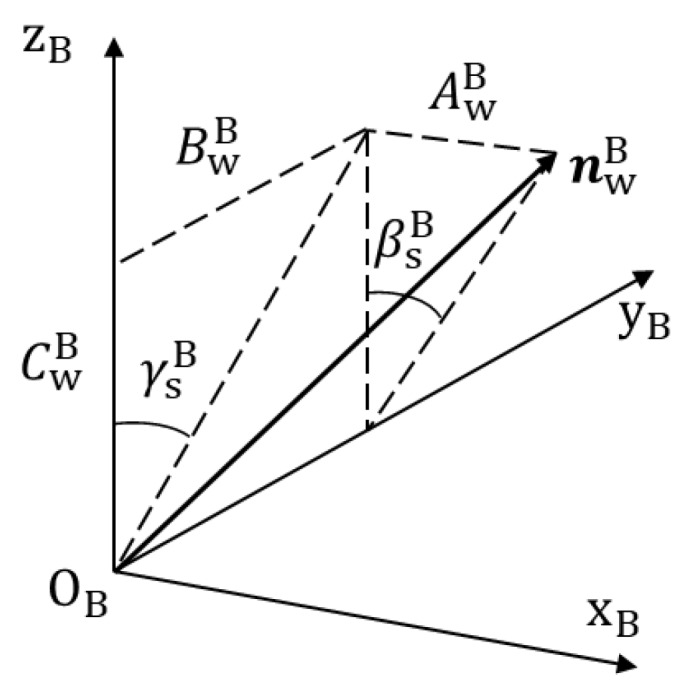
Illustration of $\gamma_{s}^{B}$ and $\beta_{s}^{B}$.

Therefore, the orientation of the joint point in {B} can be described with the Euler angles, $\gamma_{s}^{B}$ and $\beta_{s}^{B}$, as shown in Figure 12, which are given as follows:

(14) $$\{\begin{array}{l} \gamma_{s}^{B}=\arctan\left(B_{w}^{B}/C_{w}^{B}\right)\\ \beta_{s}^{B}=\arctan\left(A_{w}^{B}/C_{w}^{B}\right) \end{array}$$

So, the position and orientation of the joint point in {B} can be represented with $\left(x_{s}^{B},y_{s}^{B},z_{s}^{B},\beta_{s}^{B},\gamma_{s}^{B}\right)$.

In the welding process, besides a relative position with the joint, the welding torch also needs to maintain a desired relative orientation with the workpiece surface to ensure the welding quality. Suppose that the welding torch should be perpendicular to the workpiece surface. Then, the axis of the welding torch, $z_{T}$, needs to be parallel with $n_{w}^{B}$. In the experiments described below, the joints are mainly along the $x_{B}$ direction and there is no sharp change of their trajectories, so the welding torch does not need to rotate around its axis, $z_{T}$. Thus, $\alpha_{T}^{B}$ is constant at 0. $\gamma_{s}^{B}$ and $\beta_{s}^{B}$ are regarded as the target values of $\gamma_{T}^{B}$ and $\beta_{T}^{B}$ in the seam tracking process, respectively. Therefore, in seam tracking, the target values of the position and orientation, $\left(x_{T}^{B},y_{T}^{B},z_{T}^{B},\beta_{T}^{B},\gamma_{T}^{B}\right)$, of the robot are that of the joint point, $\left(x_{s}^{B},y_{s}^{B},z_{s}^{B},\beta_{s}^{B},\gamma_{s}^{B}\right)$, when $\alpha_{T}^{B}$ is fixed to 0.

## 4. Experiment Setup

**Figure 13:**
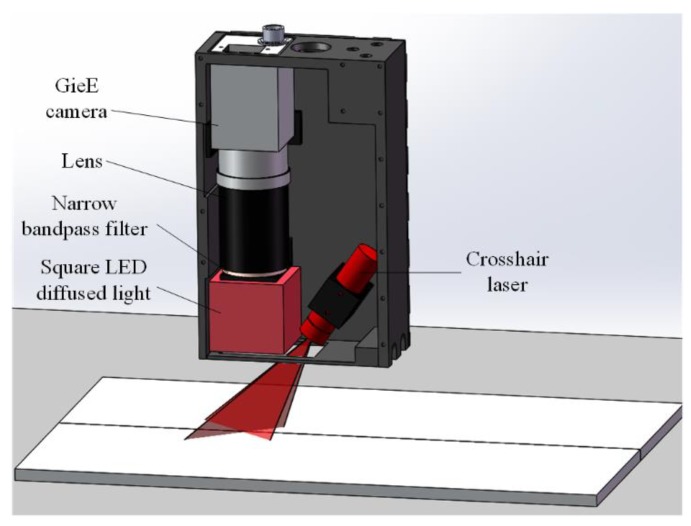
Configuration of the joint detection sensor.

The configuration of the joint detection sensor designed according to the abovementioned detection method for a narrow butt joint of GTAW is shown in Figure 13. The Gig (Gigabit Ethernet) camera has a resolution of 1600 × 1200 pixel and offers the auto exposure function. With a working distance (the distance from the bottom of the sensor to the detected workpiece) of 30 mm, the field-of-view of the camera is 20 × 15 mm. The size of the ROI is set to 1300 × 1000 pixel in the experiments. The square LED diffused light with a central wavelength of 630 nm is used as the auxiliary light source. Since the image captured by the camera is rectangular, and the shell of the sensor is cuboid, a square LED can make better use of the space in the sensor and the area in the image. The crosshair laser has a central wavelength of 635 nm and the narrow bandpass filter has a central wavelength of 635 nm and FWHM (full width at half maximum) of 10 nm. The central wavelength for the square LED diffused light, the crosshair laser, and the narrow bandpass filter can eliminate the effect of the arc light on joint detection in aluminum alloy welding using GTAW, since in the arc light spectrum, the intensity near 635 nm is relatively low [9].

**Figure 14:**
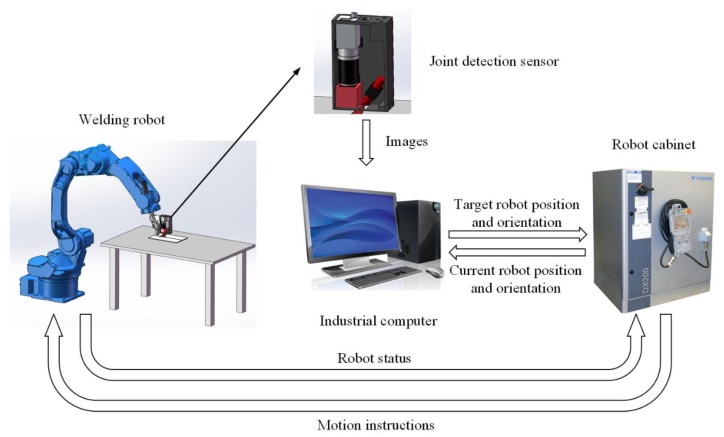
Schematic of the robotic seam tracking system for GTAW (gas tungsten arc welding).

A schematic of the robotic seam tracking system for GTAW is shown in Figure 14. The joint detection sensor is fixed 52.3 mm in front of the welding torch. The welding torch is installed at the end of the robot arm, so its position and orientation can be controlled by changing the position and orientation of the robot arm. The robot is a Yaskawa MA1440 six-axis robot, which can be controlled directly with the DX200 robot cabinet. The industrial computer has 8 G RAM and i7-6700 CPU with a clock frequency of 2.60 GHz. The image of the workpiece surface is grabbed and sent to the industrial computer by the joint detection sensor. Then, the industrial computer performs image processing to obtain the position and orientation of the joint point in the camera coordinate system {C}. By combing them with the current position and orientation of the robot sent by the robot cabinet, the industrial computer performs coordinate transformation and calculates the position and orientation of the joint point in the base coordinate system {B}, namely, the target position and orientation of the robot.

## 5. Process and Result of the Joint Detection and Seam Tracking Experiment

In this section, a joint detection experiment is carried out with the robotic seam tracking system first. Then, a seam tracking experiment is carried out and in order to smooth the detection results and improve the tracking accuracy, different smoothing methods are used and their effects are compared.

### 5.1. Process and Results of the Joint Detection Experiment

**Figure 15:**
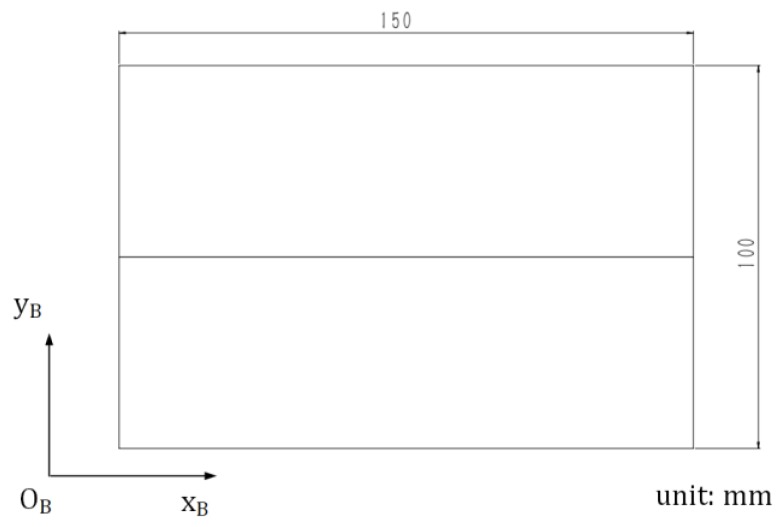
Dimension and orientation of the plane workpieces.

**Figure 16:**
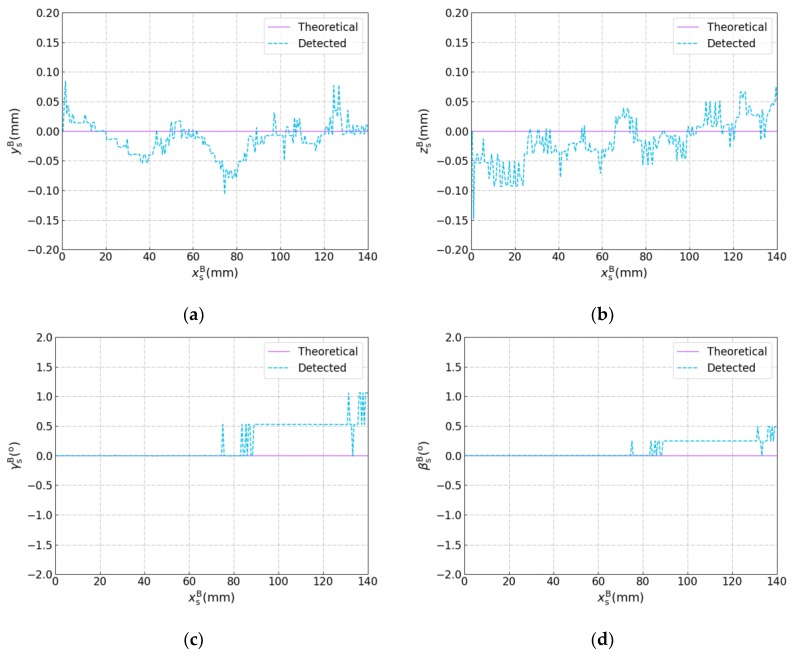
Results of the joint detection experiment with the plane workpieces. (**a**) $y_{s}^{B}$ versus $x_{s}^{B}$. (**b**) $z_{s}^{B}$ versus $x_{s}^{B}$. (**c**) $\gamma_{s}^{B}$ versus $x_{s}^{B}$. (**d**) $\beta_{s}^{B}$ versus $x_{s}^{B}$.

The joint detection experiment was performed with the robotic seam tracking system described in Section 4, in which the plane workpieces used are shown in Figure 15. The width of the joint between these two workpieces was less than 0.2 mm. The frame rate of the camera was 10 fps, and every image was used to calculate the target position and orientation. It should be noted that in this experiment, the welding torch moved along the $x_{B}$ axis of the base coordinate system {B} at a constant speed of 5 mm/s and did not change its motion status according to the detected result, so there was only joint detection and no seam tracking. The theoretical and detected results are shown in Figure 16 in which $y_{s}^{B}$, $z_{s}^{B}$, $\gamma_{s}^{B}$, and $\beta_{s}^{B}$ are plotted against $x_{s}^{B}$, respectively. The theoretical results were calculated according to drawings of the plane workpieces in Figure 15. It can be seen that the position error does not exceed ±0.15 mm and the angle error does not exceed ±1.5°, which indicates the effectiveness of the proposed detection method for narrow butt joints.

### 5.2. Process and Results of the Seam Tracking Experiment

**Figure 17:**
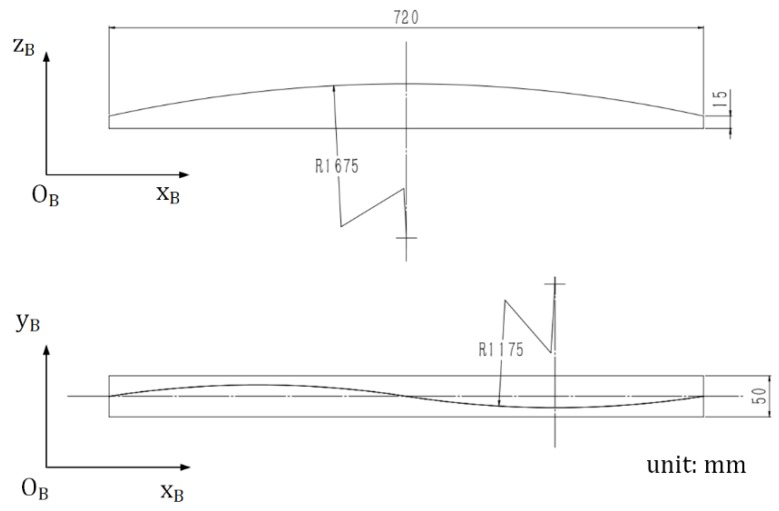
Dimension and orientation of the curved workpieces.

**Figure 18:**
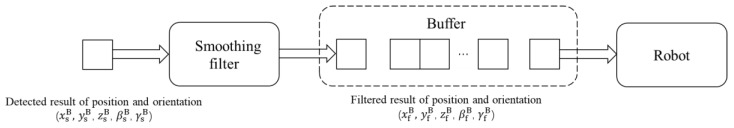
Filtering and sending of the position and orientation results.

The seam tracking experiment was performed with the robotic seam tracking system, in which the curved workpieces used are shown in Figure 17. The joint to be tracked was a curve with a 3D trajectory and width less than 0.2 mm. The frame rate of the camera was 10 fps and the linear speed and angular speed of the robot were 5 mm/s and 5 °/s, respectively. From the results of the joint detection experiment shown in Figure 16, some fluctuations can be noted. If these detected results $\left(x_{s}^{B},y_{s}^{B},z_{s}^{B},\beta_{s}^{B},\gamma_{s}^{B}\right)$ are used to guide the motion of the robot directly, dithering will happen in the robot’s motion because the detected results are not smooth enough, which will affect the accuracy of the detection and tracking. So, in the seam tracking experiment, the detected results $\left(x_{s}^{B},y_{s}^{B},z_{s}^{B},\beta_{s}^{B},\gamma_{s}^{B}\right)$ need smoothing by a filter. Then, the smoothed results of position and orientation $\left(x_{f}^{B},y_{f}^{B},z_{f}^{B},\beta_{f}^{B},\gamma_{f}^{B}\right)$ were sent to a buffer, and the results in the buffer were sent to the robot in sequence to guide its motion. The existence of the buffer is thus necessary. For the robot, we could only send it the next target after it had reached the last target. Because the next target may be detected before the robot had reached its last target, we needed the buffer to store these newly detected targets. The process is shown in Figure 18. In addition, only the filtered results whose positions were at a minimal distance (1.5 mm here) from that of the previous filtered result were sent to the buffer to make sure that positions of the filtered results used to guide the motion of the robot were not too close, otherwise obvious pauses in the motion of the robot would have resulted.

Two smoothing methods were used to smooth the detected results, and their effects were compared.

The first smoothing method was the moving average (MA). The recent 10 detected results were taken into account in order to eliminate individual results with large errors. For each dimension in these results, the maximum and minimum were excluded and the average of the rest values were calculated and regarded as the filtered result. Taking $x_{s}^{B}$ as an example, the filtered value of $x_{s}^{B}$ is denoted as $x_{f}^{B}$, which can be calculated from the following formula:

(15) $$x_{f}^{B}\left(i\right)=\frac{\sum x_{s}^{B}\left(i+k\right)-\max\left[x_{s}^{B}\left(i+k\right)\right]-\min\left[x_{s}^{B}\left(i+k\right)\right]}{8},\;k=0,1,\ldots ,9$$

where $x_{s}^{B}\left(i\right)$ is the ith value of $x_{s}^{B}$ and $x_{f}^{B}\left(i\right)$ is the ith value of $x_{f}^{B}$. For $y_{s}^{B}$, $z_{s}^{B}$, $\beta_{s}^{B}$, and $\gamma_{s}^{B}$, the same method is applied and the filtered values are calculated, respectively.

The second smoothing method was the Kalman filter (KF) [25]. The state and measurement equations for a system can be described as:

(16) $$\{\begin{array}{l} x_{i}=Ax_{i-1}+Bu_{i-1}+w_{i-1}\\ z_{i}=Hx_{i}+v_{i} \end{array}$$

where $x_{i}$ is the ith value of the variable, $w_{i}$ and $v_{i}$ are the process and measurement noise, respectively, and they are assumed to be independent, white, and with normal probability distributions, p(w)~N(0,Q) and p(v)~N(0,R), respectively, where Q is the process noise covariance and R is the measurement noise covariance. A is the state transformation matrix and B is the control matrix.

The Kalman filter iterated algorithm can be written as:

(17) $$\{\begin{array}{l} {\hat{x}}_{i}^{-}=A{\hat{x}}_{i-1}+Bu_{i-1}\\ P_{i}^{-}=AP_{i-1}A^{T}+Q\\ K_{i}=P_{i}^{-}H^{T}{\left(HP_{i}^{-}H^{T}+R\right)}^{-1}\\ {\hat{x}}_{i}={\hat{x}}_{i}^{-}+K_{i}\left(z_{i}-H{\hat{x}}_{i}^{-}\right)\\ P_{i}=\left(I-K_{i}H\right)P_{i}^{-} \end{array}$$

where ${\hat{x}}_{i}^{-}$ is the ith priori state estimate, ${\hat{x}}_{i}$ is the ith posteriori state estimate, $P_{i}^{-}$ is the ith priori estimate error covariance, $P_{i}$ is the posteriori estimate error covariance, $K_{i}$. is the ith Kalman gain, $z_{i}$ is the ith measurement, and H is the measurement matrix.

The Kalman filter was applied for each dimension of the detected results $\left(x_{s}^{B},y_{s}^{B},z_{s}^{B},\beta_{s}^{B},\gamma_{s}^{B}\right)$ to get the filtered results $\left(x_{f}^{B},y_{f}^{B},z_{f}^{B},\beta_{f}^{B},\gamma_{f}^{B}\right)$. Taking $x_{s}^{B}$ as an example, the average of the first 10 values of $x_{s}^{B}$ calculated from Equation (19) was regarded as ${\hat{x}}_{0}$. $x_{s}^{B}\left(i+10\right)$ was regarded as the measurement, $z_{i}$, so H=1. Because it was unknown and uncontrollable how the position and orientation of the joint point would change, A=1 and B=0 were set. For the other parameters, $P_{0}=0$, $Q=10^{-5}$, and R=0.01, which were determined from experience. The posteriori estimate, ${\hat{x}}_{i}$, was regarded as the filtered value of $x_{f}^{B}$; that is, $x_{f}^{B}\left(i\right)={\hat{x}}_{i}$.

**Figure 19:**
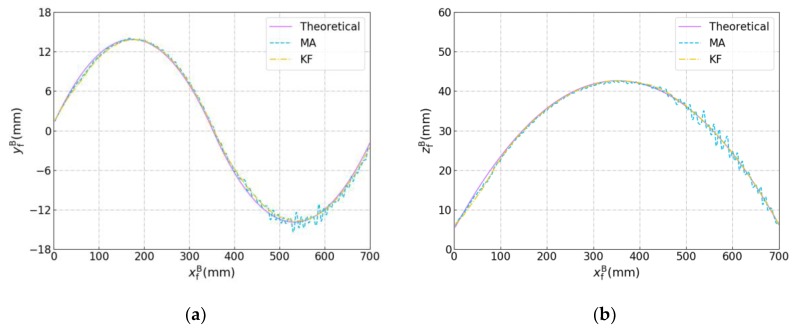

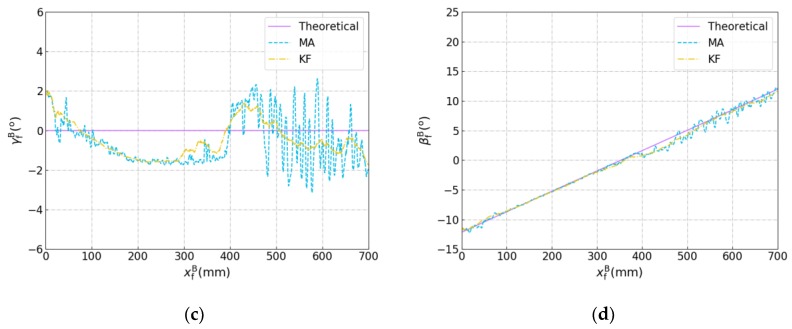
Results of the seam tracking experiment with the curved workpieces. (**a**) $y_{f}^{B}$ versus $x_{f}^{B}$. (**b**) $z_{f}^{B}$ versus $x_{f}^{B}$. (**c**) $\gamma_{f}^{B}$ versus $x_{f}^{B}$. (**d**) $\beta_{f}^{B}$ versus $x_{f}^{B}$.

The theoretical and filtered results are shown in Figure 19. The theoretical results were calculated according to the drawings of the curved workpieces in Figure 17. When MA was used, obvious dithering happens in the robot’s motion. This indicates that MA was unable to smooth the detected results effectively, so the fluctuation of the detected results caused dithering of robot’s motion as the robot performs seam tracking and its motion follows the filtered position and orientation. Because the calculation and the communication between the robot cabinet and the industrial computer need time, there was some delay (about tens of milliseconds in our experiment) between the grab of the image and the acquisition of the current position and orientation of the robot, which may bring some detection error into the coordinate transformation. When dithering starts to happen, the detection error will increase and in turn aggravate the dithering of the robot’s motion. Compared with MA, the KF can smooth the detected results and eliminate the dithering of the robot’s motion much more effectively, therefore, increasing the accuracy of joint detection and seam tracking significantly, which indicates that KF is quite applicable for the proposed robotic seam tracking system.

Next, the processing time for the joint detection and smoothing (including image processing, coordinate transformation and filtering) were tested and compared. One hundred results of the position and orientation of the joint points were detected and smoothed with MA and KF, respectively. Mean values of the required time were 44.7 ms and 44.5 ms, and the standard deviations were 9.8 ms and 8.0 ms, respectively. It can be found that KF does not lead to an increase in the processing time compared with MA. Suppose the welding speed is 10 mm/s, the distance between two detected points is less than 0.5 mm, so the processing speed of the proposed joint detection method meets the real-time requirements to make the detected trajectory accurate enough.

## 6. Conclusions

A vision based detection method for a narrow butt joint was proposed in this paper. The proposed method can detect the 3D position of the narrow butt joint with a width of less than 0.2 mm and the normal vector of the workpiece surface simultaneously. The position error does not exceed ±0.15 mm and the angle error does not exceed ±1.5 °. In addition, the proposed detection method was applied in a robotic seam tracking system for GTAW. It was found that the Kalman filter can reduce the dithering of the robot and improve the tracking accuracy significantly compared with the moving average method, which indicates that KF is applicable for the proposed robotic seam tracking system.
